# An integrative approach to unravel the *Ceratitis* FAR (Diptera, Tephritidae) cryptic species complex: a review

**DOI:** 10.3897/zookeys.540.10046

**Published:** 2015-11-26

**Authors:** Marc De Meyer, Hélène Delatte, Sunday Ekesi, Kurt Jordaens, Blanka Kalinová, Aruna Manrakhan, Maulid Mwatawala, Gary Steck, Joannes Van Cann, Lucie Vaníčková, Radka Břízová, Massimiliano Virgilio

**Affiliations:** 1 Royal Museum for Central Africa, Invertebrates Section and JEMU, Leuvensesteenweg 13, B3080 Tervuren, Belgium; 2 CIRAD, UMR PVBMT, 7 ch de l’IRAT, 97410 Saint-Pierre, La Réunion, France; 3 International Centre of Insect Physiology and Ecology (icipe), P.O. Box 30772 – 00100, Nairobi, Kenya; 4 Institut of Organic Chemistry and Biochemistry, Academy of Sciences of the Czech Republic, Prague; 5 Citrus Research International, PO Box 28, Nelspruit 1200, South Africa; 6 Sokoine University of Agriculture, Dept. of Crop Science and Production, Morogoro, Tanzania; 7 Florida Department of Agriculture and Consumer Services, Gainesville FL, USA; 8 University of Antwerp, Groenenborgerlaan 171, B-2020 Antwerp, Belgium; 9 University of Jyväskylä, PO Box 35, 40014 Finland; 10 Laboratório de Ecologia Química, Instituto de Química e Biotecnologia, Universidade Federal de Alagoas, Av. Lourival de Melo Mota, s/n, Tabuleiro, 57072-970, Maceió, AL, Brazil; 11 Institute of Chemical Technology in Prague, Technická 5, CZ-166 28 Prague 6, Czech Republic

**Keywords:** Taxonomy, *Ceratitis rosa*, *Ceratitis fasciventris*, *Ceratitis anonae*, Africa, fruit fly

## Abstract

This paper reviews all information gathered from different disciplines and studies to resolve the species status within the *Ceratitis* FAR (*Ceratitis fasciventris*, *Ceratitis anonae*, *Ceratitis rosa*) complex, a group of polyphagous fruit fly pest species (Diptera, Tephritidae) from Africa. It includes information on larval and adult morphology, wing morphometrics, cuticular hydrocarbons, pheromones, microsatellites, developmental physiology and geographic distribution. The general consensus is that the FAR complex comprises *Ceratitis anonae*, two species within *Ceratitis rosa* (so-called R1 and R2) and two putatitve species under *Ceratitis fasciventris*. The information regarding the latter is, however, too limited to draw final conclusions on specific status. Evidence for this recognition is discussed with reference to publications providing further details.

## Introduction

### Historical background and systematic position

*Ceratitis* MacLeay is an Afrotropical genus of tephritid fruit flies comprising close to 100 species found in Sub-Saharan Africa and the islands of the Western Indian Ocean. Phylogenetically it belongs to the subtribe Ceratitidina within the tribe Dacini. The latter tribe includes all main pest genera occurring naturally in Africa, i.e. *Bactrocera* Macquart, *Capparimyia* Bezzi, *Ceratitis*, *Dacus* Fabricius, *Neoceratitis* Hendel and *Trirhithrum* Bezzi. However, the monophyly of the genus is not supported and some species appear phylogenetically more closely related to *Trirhithrum*, while other *Ceratitis* species probably do not belong to the genus (Barr and McPheron 2006, Virgilio et al. 2015). Contrary to other indigenous African fruit fly pest groups that have a particular host niche width attacking particular plant families, species of the genus *Ceratitis* demonstrate a very variable host specificity. Some species are largely monophagous, while others form monophyletic stenophagous clusters specialized on particular host genera. Yet, others are polyphagous species attacking a wide variety of unrelated plants (De Meyer et al. 2002, Erbout et al. 2011). For example, the Mediterranean fruit fly, *Ceratitis capitata* (Wiedemann), is the most extreme case of polyphagy, with 304 different host plants belonging to 154 genera in 57 families considered to be suitable host plants (Liquido et al. 2014).

**Figure 1.**
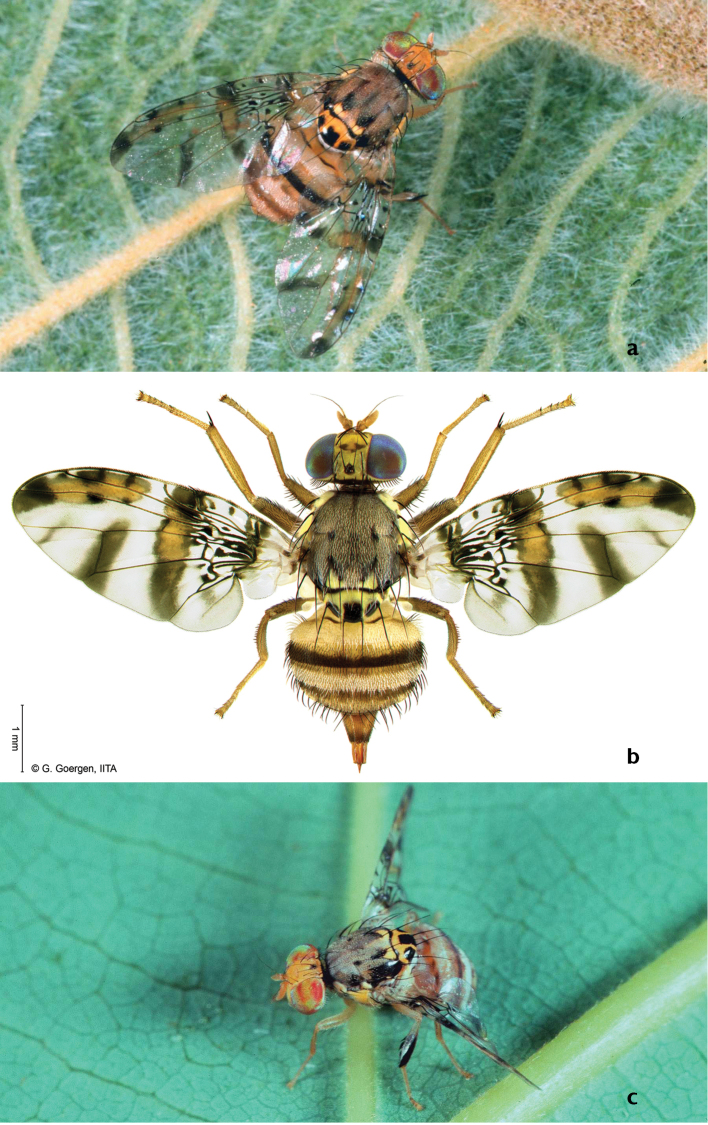
Habitus image of **a** *Ceratitis fasciventris* **b** *Ceratitis anonae* **c** *Ceratitis rosa* (**a**, **c** photos R.S. Copeland, **b** photo G. Goergen).

The *Ceratitis* FAR complex is a group of polyphagous species comprising three morphologically similar species: *Ceratitis fasciventris* (Bezzi), *Ceratitis anonae* Graham and *Ceratitis rosa* Karsch (Figure 1). Although the FAR complex is recognized as a group of closely related species, their monophyly is not always supported in phylogenetic studies. Barr and Wiegmann (2009) presented a molecular analysis for a number of *Ceratitis (Pterandrus)* species including one specimen for each *Ceratitis* FAR complex species. They state that CAD1 and ND6 markers supported the monophyly of the complex, while *tango* and *period* markers placed the three species in different clusters within the so-called ‘Pterandrus A’ group. A majority rule Bayesian tree for the combined molecular data supported the monophyly of the complex. Yet, a cladistics analysis based on adult morphological characters could not recognize them as a monophyletic cluster within the subgenus *Pterandrus* (De Meyer 2005).

### Current taxonomic status of recognized species and sexual dimorphism

The Natal fruit fly, *Ceratitis rosa*, was originally described from ’Delagoabai’ (nowadays Baia de Maputo, Mozambique and probably referring to a location near Maputo) by Karsch in 1887, based on a single male specimen (Karsch 1887). Bezzi (1920) described *fasciventris* as a variety of *Pterandrus rosa*, based upon material from Uganda (unknown locality). De Meyer (2001) considered it to be a separate entity with specific status. *Ceratitis anonae* was described by Graham from material reared from an *Annona* fruit in Ashanti, Ghana (Graham 1908).

**Figure 2.**
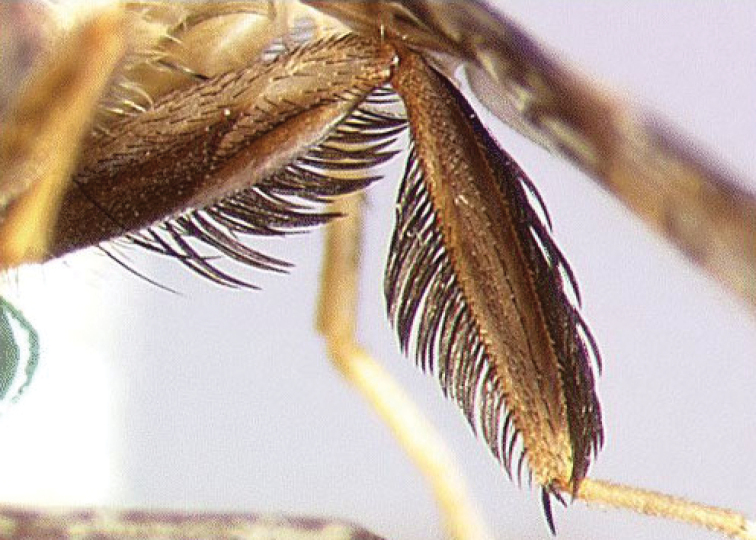
Mid leg of male *Ceratitis anonae*, anterior view of femur and tibia (photo I.M. White, NHM).

**Figure 3.**
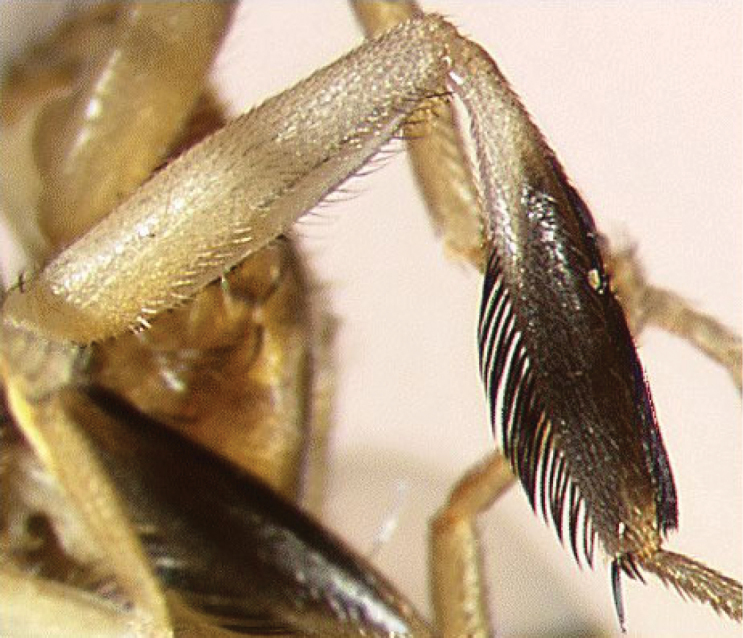
Mid leg of male *Ceratitis rosa*, anterior view (photo I.M. White, NHM).

**Figure 4.**
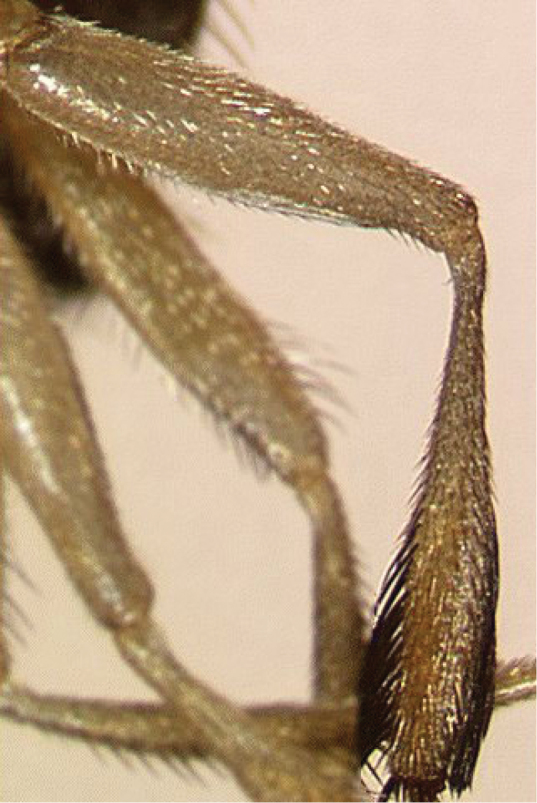
Mid leg of male *Ceratitis fasciventris*, anterior view (photo I.M. White, NHM).

All three species show remarkable sexual dimorphism, with the males having leg ornamentation that is absent in females. *Ceratitis anonae* males have the mid leg (Figure 2) with a row of long dark, flattened setae (so-called ‘feathering’) ventrally along the entire length of the mid femur. The mid tibia is broadened with feathering dorsally along distal 0.9 and ventrally along the distal 0.8. The mid leg is largely brownish to brownish black in colour. In *Ceratitis rosa* males (Figure 3), the ventral feathering on mid femur is absent (at most there are a few thin and dispersed setulae ventrally). The mid tibiae is moderately broadened, anteriorly black with a conspicuous silvery reflection seen when kept under a certain angle and black feathering dorsally along distal 0.75 and ventrally along distal 0.66–0.75. Otherwise, the leg is yellow. *Ceratitis fasciventris* males have the mid leg shaped similarly to *Ceratitis rosa*, except that the mid tibia is not distinctly broadened and the black feathering is restricted to the distal 0.5 at most (Figure 4). The leg is coloured uniformly yellow, except in some specimens where the anterior part is partially brownish in the distal 0.3 (De Meyer 2001). Preliminary courtship behavior studies by Quilici et al. (2002) give some indication that, for at least in *Ceratitis rosa*, the mid legs are used in precopulatory behavior.

Unlike the males, females, are morphologically almost indistinguishable. *Ceratitis anonae* females differ from the other two taxa in the pilosity of the anepisternum and fore femur. In *Ceratitis anonae* the anepisternum has some few dark setulae medioventrally, and the fore femur has dispersed short dark setulae between the ventral setae and posterior row of setae. In *Ceratitis rosa* and *Ceratitis fasciventris*, the anepisternal pilosity is completely pale and the fore femur usually only has pale setulae present between the ventral setae and posterior row of setae (De Meyer and Freidberg 2006). Females of *Ceratitis rosa* and *Ceratitis fasciventris* females cannot be reliably differentiated on morphological characters.

### Distribution patterns throughout Africa

**Figure 5.**
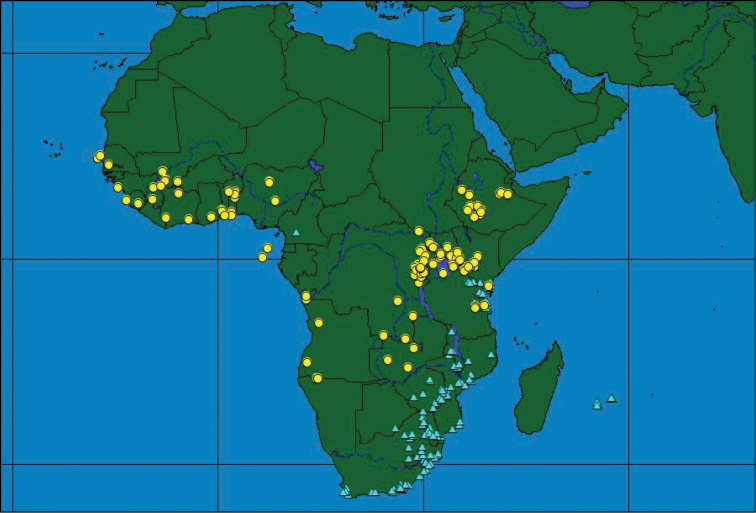
Geographical distribution of *Ceratitis rosa* (blue triangles) and *Ceratitis fasciventris* (yellow circles).

**Figure 6.**
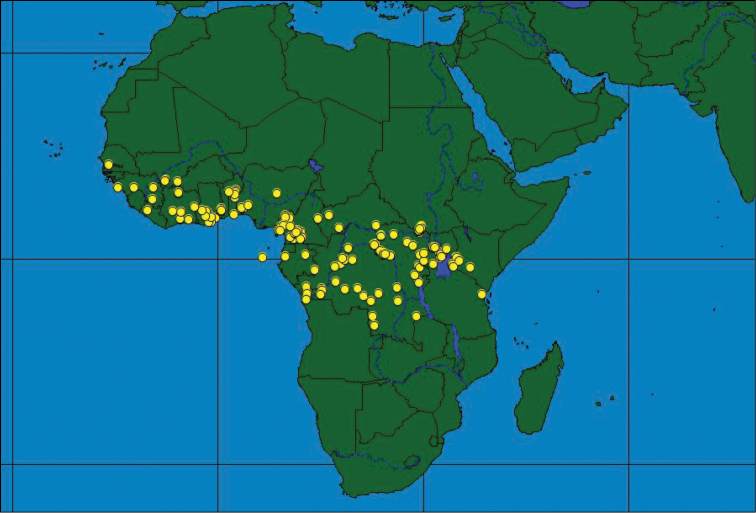
Geographical distribution of *Ceratitis anonae*.

The occurrence of the three species throughout Africa shows different distribution patterns with only a partial overlap between some of the species. Although some species do occur sympatrically or parapatrically, nowhere do all three species co-occur. *Ceratitis rosa* is found throughout South (from western Cape onwards) to East Africa with the northernmost records from the Central Highlands in Kenya (Figure 5). The latter occurrence seems to be a recent expansion (Copeland and Wharton 2006) with its previous extension reaching till the Kenyan coast only. It has been introduced to the Indian Ocean Islands of La Réunion and Mauritius in the 20^th^ Century (White et al. 2000). The single record of *Ceratitis rosa* (one male) from Cameroon in West Africa is probably due to mislabeling, while the presence of the species in Ivory Coast (N’dépo et al. 2010, 2013) is an erroneous record. No reliable records from western Africa have been found. *Ceratitis fasciventris* has a wider distribution and is found throughout western Africa, with isolated records from Central Africa, and extensive distribution along the Albertine and Gregory Rifts in eastern Africa, as far north as Ethiopia (Figure 5). *Ceratitis anonae* has a predominantly equatorial belt distribution, being widespread through West (from Senegal onwards) and Central Africa (Figure 6). Its easternmost distribution seems to be confined to the western side of the Gregory Rift in Kenya (Copeland et al. 2006) (records further to the east need confirmation).

Sympatric and allopatric occurrence is found between *Ceratitis anonae* and *Ceratitis fasciventris* in western Africa (Vayssières et al. 2004), and western Kenya (Copeland and Wharton 2006), while *Ceratitis rosa* and *Ceratitis fasciventris* occur together in parts of Kenya and Tanzania (Figure 5) (Copeland et al. 2006, Mwatawala et al. 2006). *Ceratitis anonae* and *Ceratitis rosa* were not found together.

### Indications of inconsistencies

Despite the morphological differences in adult males and the partially disjunct distribution, there are indications that cryptic speciation occurs within the three currently recognized morphospecies.

When comparing sequences of mitochondrial and nuclear markers, Virgilio et al. (2008) could not recover the three morphospecies as monophyletic groups, although different markers recovered well-supported clades within the representatives of *Ceratitis fasciventris* from West and East Africa. So, although the molecular data did not contradict or support the morphological separation, it did suggest that *Ceratitis fasciventris* is itself a complex of cryptic species. This study, as well as Douglas and Haymer (2001) and Barr et al. (2006) indicated moreover the existence of other separate clusters within the complex (although not always with high support).

Finally, correlative ecological niche modeling showed that *Ceratitis rosa* prefers climatic conditions with lower temperatures when compared to *Ceratitis capitata* (De Meyer et al. 2008). This was corroborated by findings on the island of La Réunion where *Ceratitis rosa* occupies a colder and more humid climate niche than *Ceratitis capitata* (Duyck et al. 2006). However, these results were contradicted by Grout and Stoltz (2007) who studied the developmental thresholds for a number of *Ceratitis* species, based on South African populations and concluded that the situation in South Africa is different from that in La Réunion. This led to the suggestion that *Ceratitis rosa* could comprise entities with different biological requirements.

### Objective

The inclusion of the *Ceratitis* FAR complex in the Coordinated Research Project (CRP) on cryptic species, therefore, was to establish whether the three morphological entities actually represent three distinct species, or if the relationship can be resolved in a different manner. The authors opted for an integrative approach, using different methodologies, including population genetics, larval morphology, wing morphometrics, cuticular hydrocarbons, developmental physiology and pre- and postzygotic mating compatibility. The results of these different approaches are presented in this volume or have been published elsewhere (Virgilio et al. 2013, Vaníčková et al. 2014). We herewith summarize the main results obtained by each of these approaches and provide a synthesis on the current knowledge and status of the different entities within the *Ceratitis* FAR complex.

## Results

### Microsatellite analysis

**Figure 7.**
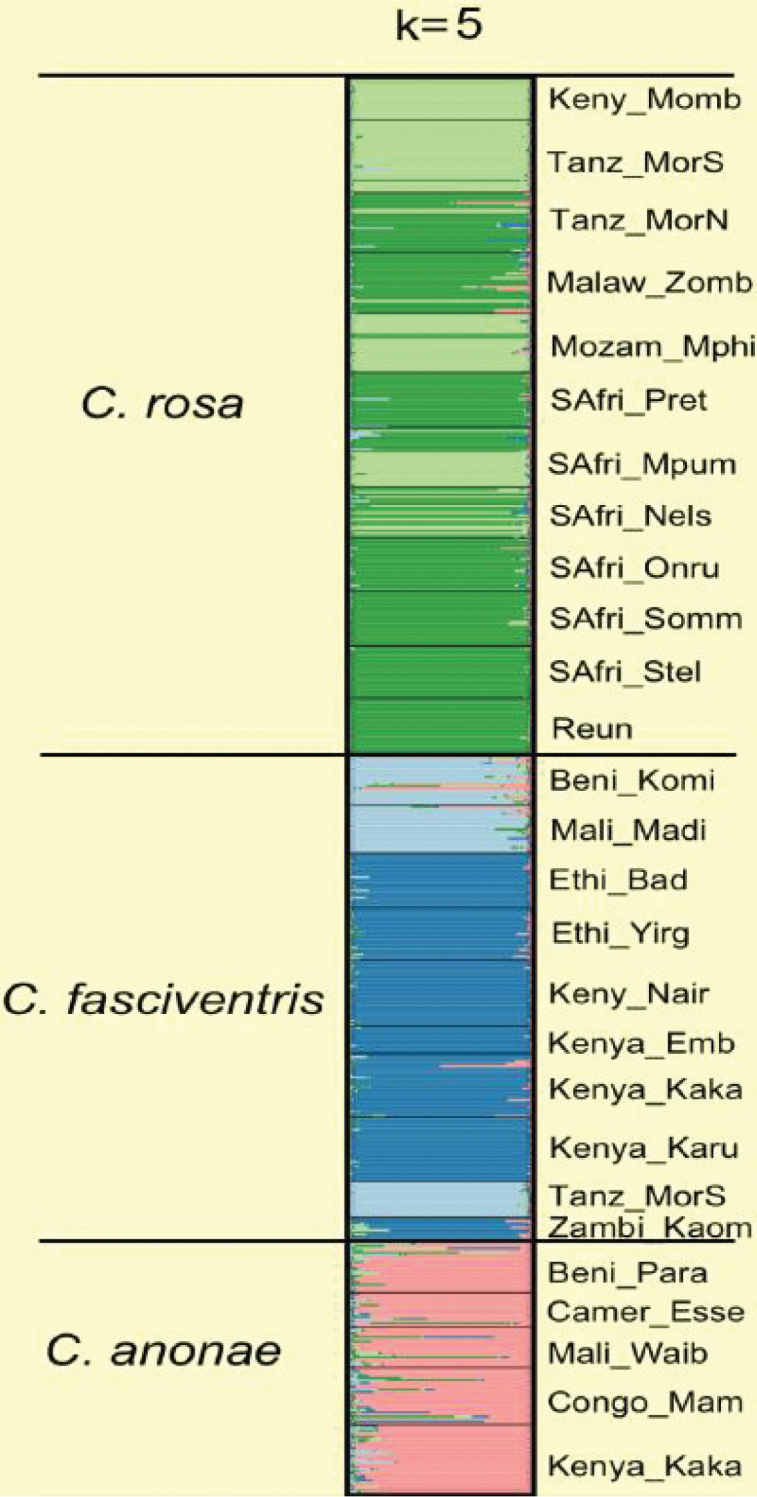
Structure of admixture proportions of 621 specimens of the FAR complex, assigned to five genotypic clusters (from Virgilio et al. 2013).

Using microsatellites, Baliraine et al. (2004) revealed significant differentiation with respect to genotypic frequencies between *Ceratitis rosa* specimens from the African mainland and the Indian Ocean islands, and between *Ceratitis fasciventris* from Kenya and from Uganda. Delatte et al. (2013) isolated and characterized 16 microsatellite markers using representatives of the three species from 11 localities in Africa. Subsequently, Virgilio et al. (2013) used these microsatellites to survey the allelic variation in 27 African populations of the three morphospecies. Their main finding was that the complex comprises five genotypic clusters, based upon the individual Bayesian assignments of STRUCTURE (Pritchard et al. 2000): a single *Ceratitis anonae* genotypic cluster, two clusters for *Ceratitis fasciventris* (allo- and parapatric) and two clusters, of *Ceratitis rosa* (allo- and sympatric) (Figure 7). The sympatric *Ceratitis rosa* clusters were observed in two South African sampling sites (from a sample coverage including *Ceratitis rosa* from Malawi, Mozambique, Kenya, la Réunion and Tanzania). The two *Ceratitis fasciventris* genotypic clusters roughly corresponded to a western African and an eastern African group, except for a single population from Tanzania that was more genetically similar to the West African samples. The two phylogenetic *Ceratitis fasciventris* clades (Virgilio et al. 2008) corresponded to the two microsatellite genotypic clusters. Unfortunately, voucher material of Baliraine et al. (2004) could not be traced (Malacrida, personal communication) so that their microsatellite data could neither be fitted into the patterns observed in Virgilio et al. (2013) nor used for morphological analysis. The results of the microsatellite analysis were used to corroborate the morphospecies hypotheses and to generate new hypotheses which were then further tested through an integrative taxonomical approach.

### Adult morphology

**Figure 8.**
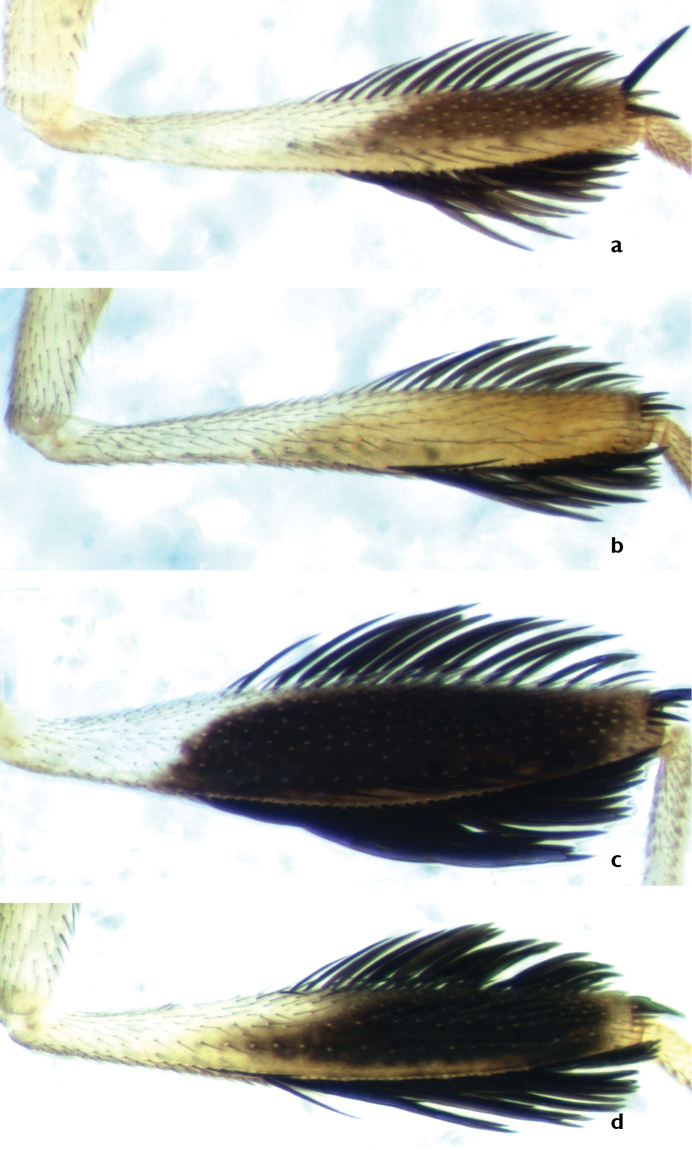
Anterior view of male mid tibia in *Ceratitis fasciventris* (**a** F1, **b** F2) and *Ceratitis rosa* (**c** R1, **d** R2).

The 399 male specimens of the 27 African populations used for the microsatellite analysis were, in light of the above findings, re-examined. Preliminary observations indicated a difference for *Ceratitis fasciventris* in the coloration of the male mid tibia with specimens from western Africa demonstrating darker coloration (De Meyer 2001) (henceforth referred to as “F1”; Figure 8a), whereas specimens from eastern populations have completely yellow mid tibia (“F2”; Figure 8b).

Re-examination of the *Ceratitis rosa* specimens revealed slight morphological differences with one type bearing a broader and more stout mid tibia with black coloration reaching the lateral margins of the tibia throughout (“R1”; Figure 8c), while the other morphotype bearing a more slender tibia, gradually tapering towards the base, and with the black coloration not reaching the lateral margins throughout the full length (“R2”; Figure 8d).

All males were scored blindly (i.e. without reference to the genotypic cluster they belong to) for these morphological characters. Morphological assignment for R2 and F2 was 100% in alignment with the genotypic clustering while R1 and F1 had, respectively, 3.4% and 10% uncertain assignment (i.e. could not be assigned unambiguously to any of the genotypic clusters) because of doubtful interpretation of the morphological character. None of the specimens examined scored contradictorily to the genotypic cluster. It was concluded that the morphological differences observed in the male mid leg shape and coloration are a reliable character state when combined with genotyping to differentiate the different populations of *Ceratitis rosa* and *Ceratitis fasciventris*.

### Wing morphometrics

Since no morphological characters were found to reliably differentiate females neither of the different morphospecies nor of the five genotypic clusters recognized by the microsatellite analysis, a morphometric study was conducted on the wings of the same specimens used for the microsatellite (males and females) and the adult morphology studies (males only). Two techniques were used by Van Cann et al. (2015): landmarking based on intersections between wing veins and cross-veins, and measurement of wing band areas. In total 227 specimens, previously morphologically identified and genotyped at 16 microsatellite loci, were used. Seventeen wing landmarks and six wing band areas were used for morphometric analyses, mainly from unambiguous points such as intersections between veins and cross-veins, or between the former and bands. Significant differences were found both among the three morphospecies as well as among the five genotypic clusters when Permutational Multivariate Analysis of Variance (PERMANOVA) was conducted. Unconstrained and constrained ordinations could not resolve these groups but posterior group membership probabilities (PGMPs) of the Discriminant Analysis of Principal Components (DAPC) showed that wing landmarks (and, to a less extent, wing band areas) could be used to consistently assign a relevant proportion of specimens to morphospecies (range 96.8–98.2%) and genotypic cluster (range 87.5–96.4%). This indicates that wing morphometrics does reflect to a certain extent the clustering and division in different genotypic clusters.

### Larval morphology

Recognition of larval stages of fruit fly pests is a necessity given the fact that most quarantine interceptions are larvae inside imported fruits during regulatory checkpoints. However, identification of fruit fly larvae at species level is notoriously difficult because of the limited morphological characters and the high intraspecific variability in character states. The study by Steck and Ekesi (2015) on representatives of the *Ceratitis* FAR complex and comparison with *Ceratitis capitata*, has confirmed the difficulties in this matter. Among the different populations studied of *Ceratitis fasciventris* (1), *Ceratitis anonae* (1) and *Ceratitis rosa* (5, including the two genotypic clusters from different geographical regions, i.e. Kenya and South Africa), only larvae of *Ceratitis fasciventris* could be unambiguously distinguished from the other species of the FAR complex, based on differences in quantitative measures of numerous larval morphological characters (at least as observed in the single population studied). Although there was also variation observed in diagnostic morphological characters among the larvae of the different *Ceratitis rosa* populations studied, the two genotypic clusters (R1 and R2) could not be differentiated consistently. Moreover, characters once considered reliable to separate the larvae of *Ceratitis capitata* from *Ceratitis rosa* (Carroll, 1998), or even to separate the genus *Ceratitis* from the genus *Bactrocera* (White & Elson-Harris, 1992) have shown to be variable among the different species and populations studied here and not diagnostic for a particular taxon.

### Cuticular hydrocarbons

**Figure 9.**
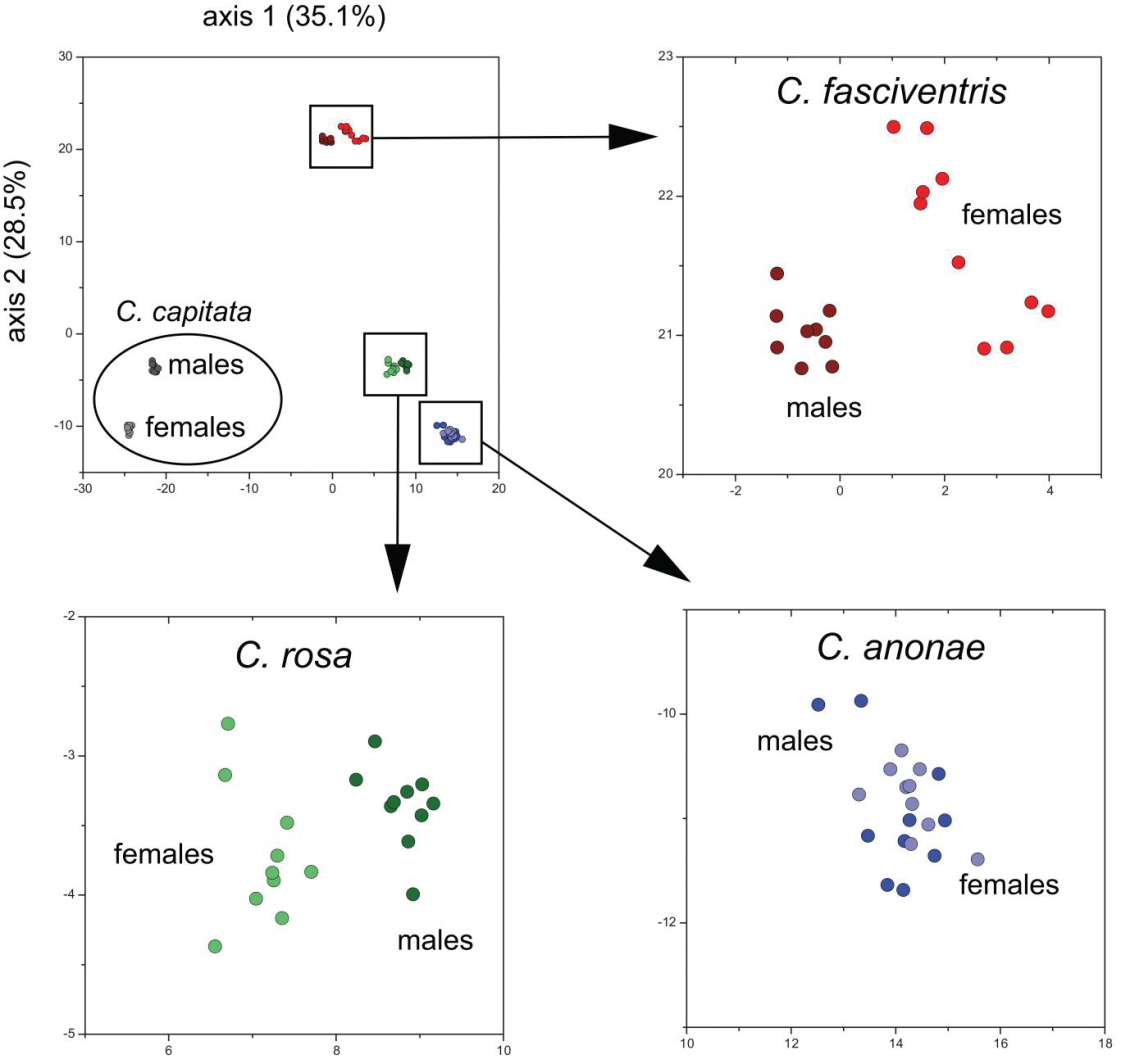
Principal components analysis (PCA) of Euclidean distances between males and females *Ceratitis capitata*, *Ceratitis fasciventris*, *Ceratitis anonae* and *Ceratitis rosa* (as calculated from peak areas of 59 cuticular hydrocarbons (CHCs)).

Cuticular hydrocarbons (CHCs) comprise a majority of the components of the cuticular waxes in many insects and may include *n*-alkanes, *n*-alkenes, terminally monomethylalkanes, dimethylalkanes among others (Blomquist and Jackson 1979). The long-chain CHCs play central roles in waterproofing of the insect cuticle and function extensively in chemical communication as sex pheromones, and species and sex recognition cues among others (Blomquist and Bagnères 2010). As a result of their species-specificity, CHCs are widely used for identification of sibling or cryptic species (Kather and Martin 2012). Recent studies on CHC profiles of drosophilid (Jennings et al. 2014) and tephritid flies (Vaníčková 2012, Vaníčková et al. 2015) evaluated the use of CHCs in delineating groups within supposedly cryptic taxa. An initial analysis included one population each of the three morphospecies within the FAR complex and *Ceratitis capitata* as a comparative taxon (Vaníčková 2012, Vaníčková et al. 2014). Male and female specimens were obtained from the colonies kept at the International Centre for Insect Physiology and Ecology, ICIPE (for FAR complex) and the FAO/International Atomic Energy Agency Agriculture and Biotechnology Laboratories in Seibersdorf (for *Ceratitis capitata*) and were genotyped, following Delatte et al. (2013) and Virgilio et al. (2013). The *Ceratitis rosa* population represented the R2 genotypic cluster, while the *Ceratitis fasciventris* population represented the F2 genotypic cluster. Using two-dimensional gas chromatography with mass spectrometric detection, differences in the CHC profiles between all three species (but also between sexes) were observed with twelve compounds indicated as possible chemotaxonomical markers to differentiate the three types (Figure 9). After recognition of the two *Ceratitis rosa* genotypic clusters (cf above), the analysis was repeated for one population each of the R1 and R2 type obtained from ICIPE colonies (Vaníčková et al. 2015b). This resulted in four potential markers that allow differentiation between the R1 and R2 genotypic clusters. CHCs as such provide indication of qualitative differentiation in their profiles to allow distinction between at least four of the five genotypes (the F1 genotype could not be included because of lack of an established colony that could provide material for analysis). These findings should preferably be further explored by using populations from other regions for the same genotypes as there is evidence that also elements such as diet can have an impact on CHC intraspecific variability.

### Pheromones

Pheromones in investigated FAR complex species are produced exclusively by males and are, similarly as in other fruit fly species, highly complex species-specific mixtures characterized by specific qualitative and quantitative profiles of diverse chemical structures, including alcohols, aldehydes, terpenes, and esters. GCxGC-TOFMS analysis of male *Ceratitis* FAR complex pheromones observed in single population samples for each species, resulted in the identification of 35 compounds produced by *Ceratitis fasciventris* F2 type, 18 compounds released by *Ceratitis anonae* and 26 volatiles emitted by *Ceratitis rosa* R2 type. The composition of male pheromones partially overlapped, but contained also species-specific chemical compounds. Only 12 compounds elicited antennal responses suggesting a prominent role in pheromone communication. Four of the compounds were found in emanations of all three studied species (Břizová et al. 2015). However these shared compounds are present in the respective species in different concentrations. Electrophysiological data showed that females of the *Ceratitis* FAR complex species perceive components of conspecific pheromones specifically, e.g. they are specifically tuned to conspecific pheromones, but antennae respond also to pheromone components of other species. Both males and females can perceive male pheromones. However to decide whether there are sex-specific differences, further electrophysiological experiments are needed.

Many volatiles identified in *Ceratitis* FAR male emanation have been previously identified in other tephritid pheromones (Cruz-López et al. 2006, Heath et al. 1991, Lu and Teal 2001, Milet-Pinheiro et al. 2014, Robacker 1988, Rocca et al. 1993, Vaníčková 2012, Vaníčková et al. 2012). Others, specifically isoprenoid geranyl acetone, aliphatic volatile (*E*)-non-2-enal, and methyl (2*E*,6*E*)-farnesoate, have not been reported before. Interestingly, the latter is a crustacean reproductive hormone, structurally similar to insect juvenile hormone, which is responsible for enhancing reproductive maturation, maintaining juvenile morphology, and influencing male sex determination (Olmstead and LeBlanc 2007, Nagaraju and Borst 2008). In insects, methyl (2*E*,6*E*)-farnesoate represents the immediate precursor of insect juvenile hormone III (Teal et al. 2014). As a semiochemical, methyl (2*E*,6*E*)-farnesoate was reported in pentatomid bug pheromones (Millar et al. 2002).

The pheromone composition as well as electroantennography may be used for species identification. Similarly as for the composition of CHCs (Vaníčková et al. 2014), the pheromone composition and antennal specificity suggest that the three nominal species of the *Ceratitis* FAR complex correspond to taxonomically well-defined entities. It is recommended, however, that this work should be expanded by including pheromone studies of F1 and R1 and further sampling and analysis of different populations for all types, to determine inter-population variability.

### Developmental physiology

Developmental physiology studies can assist in detecting differences between species with regard to biological requirements. Only the developmental physiology of the two *Ceratitis rosa* types was studied in detail (Tanga et al. 2015), because of the lack of established colonies for the other species and because it was the differences observed between developmental studies conducted in La Réunion (Duyck and Quilici 2002) and South Africa (Grout and Stolz 2007) that initiated the idea that *Ceratitis rosa* may consist of different biotypes with different climatic requirements. R1 and R2 populations were, therefore, studied simultaneously in Kenya and South Africa (Tanga et al. 2015). Depending on locality and temperature, marked differences were observed between R1 and R2 populations in the developmental duration of immature life stages. In both Kenyan and South African populations, R2 appears to be less adapted to hotter environments than R1. In Kenya, R2 appeared to be better adapted to colder environments, while in South Africa, both *Ceratitis rosa* types were able to tolerate lower temperatures. Despite discrepancies in temperature related developmental physiology of the two types in the two localities, results from Kenya and South Africa clearly demonstrate and support the existence of two genetically distinct populations of *Ceratitis rosa* that are divergent in their physiological response to temperature. Discrepancies between the localities with regards to observed and estimated developmental time parameters of the two *Ceratitis rosa* types can be due to geographic variation (populations from South Africa originated from temperate climates and populations from Kenya originated from tropical climates). Other factors such as food quantity and quality, rearing conditions, acclimatization and generation age are also mentioned as factors influencing the developmental physiology.

### Geographical distribution and altitudinal transect

**Figure 10.**
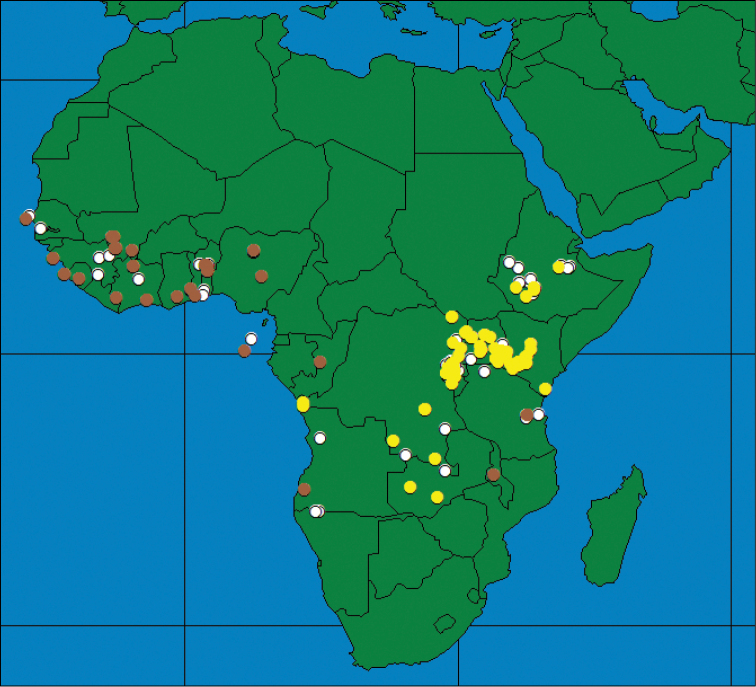
Geographical distribution of *Ceratitis fasciventris*, F1 (brown circles), F2 (yellow circles), un-assigned (white circles).

**Figure 11.**
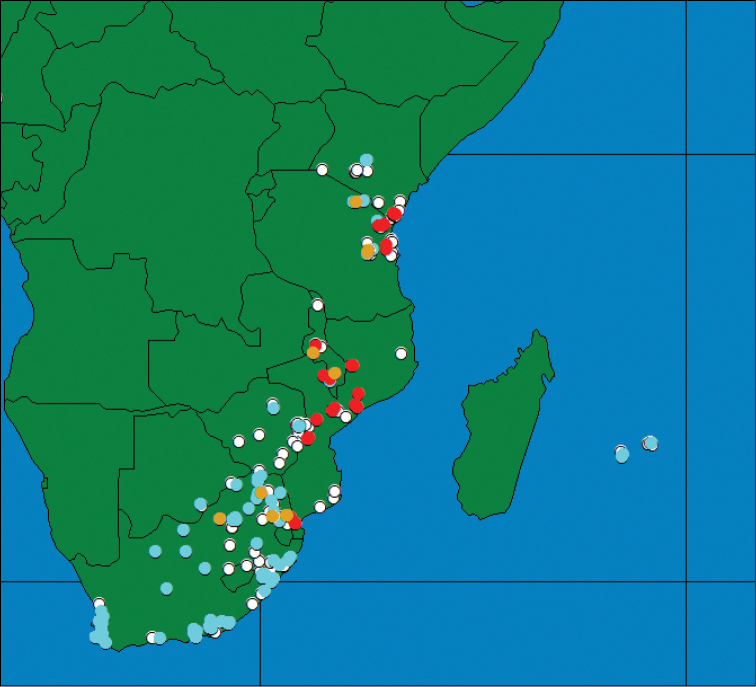
Geographical distribution of *Ceratitis rosa*, R1 (red circles), R2 (blue circles), sympatric occurrence (brown circles), un-assigned (white circles).

The distribution of *Ceratitis fasciventris* and *Ceratitis rosa* was re-analyzed, taking into account the existence of two types for each of these species. As the known distribution was largely based upon museum specimens collected over a period of 130 years and the DNA retrieval from older specimens (i.e. >10yrs) is cumbersome and with low success rate, it was decided to re-assign specimens based only on morphological characters which excluded female specimens. In total, specimens from 218 localities were re-examined and assigned to one of the four types (F1, F2, R1, R2). The observed distributions are given in Figures 10–11. F1 is mainly represented in western Africa but extends its distribution throughout southern and eastern parts of the continent with records from Angola, Zambia, Malawi and Tanzania (Figure 10). F2 is confined to the Rift areas of Ethiopia, Kenya and eastern part of the Democratic Republic of Congo, with southern expansion into Katanga region of the Democratic Republic of Congo and Zambia. So far, no sympatric or parapatric occurrence of the two types has been observed. R1 and R2 on the other hand do not show a clear geographic isolation (Figure 11). Only in the Cape and central parts of South Africa is a single type (R2) present, as well as in the adventive populations on the Indian Ocean islands. In the northern part of South Africa and northwards there are records showing overlap of distributions.

A detailed study was conducted in the Uluguru Mountains near Morogoro (Tanzania) where both types are known to occur (Mwatawala et al. 2015). Along an altitudinal transect, ranging from 540 to 1650 masl the occurrence of both types was monitored using traps with EGO-lure. A gradual shift was observed with both types occurring at lower altitudes (with predominance of R1) while only R2 was observed at the highest elevations. Geurts et al. (2012) observed a temperature shift of 7–8 °C along this transect. When looking at the developmental differences observed for, at least the Kenyan populations, temperature could well play a major role in the observed pattern, although it remains to be seen whether other aspects such as differential host range and availability also have an influence regarding the differences observed.

### Pre- and postzygotic incompatibility

Although no detailed data are presented in this volume, preliminary data by S. Ekesi (pers. communication), indicate that in field cage studies there is a significant pre- and post-zygotic incompatibility both between *Ceratitis rosa* and *Ceratitis fasciventris* (F2), as well as between the two genotypic clusters of *Ceratitis rosa*: R1 and R2. None of the other genotypic clusters or morphospecies were included.

## Conclusion

The majority of the research approaches discussed here indicate that the *Ceratitis* FAR complex consists of at least the three recognized morphospecies, but possibly of five different species. All methodologies (except larval morphology) used confirm that *Ceratitis anonae*, *Ceratitis fasciventris* and *Ceratitis rosa* are well recognized groups. *Ceratitis anonae* is a uniform group showing no apparent morphological or genetic variability and has a well-defined distribution range. The two other entities consist each of two separate groups. For *Ceratitis rosa*, the two entities (called ‘R1’, ‘lowland’ or ‘hot rosa’ on one hand, and ‘R2’, ‘highland’ or ‘cold rosa’ on the other hand) can be distinguished morphologically in males, as well as by other means and demonstrate a different developmental physiology. They occur sympatrically in some regions, but also show a disjunct distribution that appears to be correlated with ambient temperature. It is concluded that both types should be considered as two different species. Taxonomically, the type material of *Ceratitis rosa* belongs to the R1 type, which means that the R2 type should be considered as a new species, and a formal description will be published in the near future. Similarly, the review indicates that also *Ceratitis fasciventris* tends to be composed of two entities. Yet, because the data are currently insufficient to establish clearly whether they should also be considered two different species or not, we currently suggest to maintain the two types under one and the same species.
